# Dr. Markwick on Rhubarb as an Application to Ulcerated Surfaces

**Published:** 1842-09-24

**Authors:** 


*Rhubarb as an Application to Ulcerated Surfaces.*—Dr. ALFRED MARKWICK writes to the Editor of the London Medical Gazette that while externe at the Male Venereal Hospital, Paris, under the justly celebrated and much lamented M. Cullerier, who died some few months since of acute hepatitis, a patient was placed under his care, who had already been an inmate of the hospital upwards of two months, for severe and extensive ulceration of the abdomen, occupying the whole of its right side and greater part of its left. The right wrist was also much affected. He had unfortunately inoculated it from the abdomen. Almost every plan of treatment had been adopted; even the actual cautery had been applied three times, but without producing any arrest to the progress of the sloughing. At the time I entered the hospital (says Dr. M.) he was much emaciated; his face was expressive of great suffering and despair; he had no appetite; his spirits were very bad, so that he would sometimes cry like a child; and he passed restless nights. The application that was then used was a combination of bark and camphor, which was sprinkled lightly over the parts. I continued this for rather more than two months, and he took at the same time sarsaparilla, and iodide of mercury; but he derived no benefit: indeed he became still more emaciated, and he had a perfectly sanguineous appearance. I then suggested to M. Cullerier the employment of the powdered rhubarb. To this he readily consented; and the following morning it was applied, but only in very small quantity, on account of the violent irritation it produced. Over this was placed some linen with holes cut in it about a quarter of an inch apart, (*linge troué*, as it is called by the French,) which was covered with charpie. On removing the dressings the following morning, a decided amelioration was perceptible. The wound had assumed a much more healthy character. M. Cullerier advised some camphor to be added to the rhubarb, in order, if possible, to mitigate the pain occasioned by the application. This was done ; but it had not the desired effect. The powder was then only applied every other day for about six weeks, when the ulcers had nearly healed. His appetite, spirits, and appearance daily improved, and in about a fortnight afterwards he was able to leave the hospital quite recovered. This patient had remained in the hospital four months without obtaining any relief to his sufferings. Six weeks were sufficient to produce cicatrization of nearly the whole ulcerated surface with the rhubarb—a fact which I think sufficiently proves the efficacy of the drug in sloughing venereal ulcers.

The patient had no return of the disease when I saw him six months afterwards.

